# Comparing the Effectiveness of GLUMA and 940 nm Laser for Improving Crown Retention with Self-Adhesive Cement

**DOI:** 10.30476/dentjods.2024.99630.2165

**Published:** 2025-03-01

**Authors:** Pantea Amiri, Ghazale Tekie, Arash Azizi, Shirin Lawaf

**Affiliations:** 1 Dept. of Oral Medicine, Tehran University of Medical Sciences, Islamic Azad University, Tehran, Iran; 2 Dept. of Prosthodontic, Tehran University of Medical Sciences, Islamic Azad University, Tehran, Iran

**Keywords:** Crowns, Resin Cements, Lasers, Prosthesis Retention

## Abstract

**Statement of the Problem:**

The retention of dental crowns plays a pivotal role in their long-term success and maintenance. According to the ongoing controversy about the effect of GLUMA desensitizer and diode laser on the retention of full metal crowns, this study seeks to investigate the effectiveness of two different methods in enhancing the bond strength of full-metal crowns.

**Purpose:**

This study was developed to compare the effect of 940nm diode laser and GLUMA desensitizer on the bond strength of full-metal crowns cemented by self-adhesive resin cement (RelyX U200).

**Materials and Method:**

**Results:**

Crown retention in the 940 nm diode laser group (166.86±34.25 N) was significantly lower than the GLUMA desensitizer group (318.59±56.31 N) (*p*< 0.05), but there was no significant difference with the control group (138.17±40.81). Crown retention in the GLUMA desensitizer group was significantly higher than the other groups (*p*< 0.05)

**Conclusion:**

Results of this study showed that GLUMA desensitizer had a positive effect, and 940nm diode laser had no effect on the retention of crowns cemented by self-adhesive resin cement.

## Introduction

One of the concerns and determining factors in dental crowns remaining in place on the prepared teeth is the retention factor. The most important retention element is the presence of two opposing vertical surfaces, which can be buccal and lingual walls of full crowns [ 1 ]. It has been shown that the absence of retention is a usual factor failure of fixed prosthesis [ 2 ]. The retention of crowns depends on the taper. Based on previous studies, maximum retention has been shown to range from 6 to 12 degrees [ 3 - 4 ]. Optimal retention for extracoronal crowns depends on convergence, the surface area of the preparation, auxiliary grooves, and type of cement used [ 5 ].

If retention is not achieved, complications such as microleakage and complete removal of the crowns can occur [ 5 - 6 ]. 

Most patients experiencing fixed restorations feel irritation in the prepared teeth either before or after restoration is placed, which can be understood as pain, which may be caused by dentin hypersensitivity [ 7 ]. Dentin hypersensitivity can be explained by brief, sharp emanating from exposed dentin in reaction to different stimuli and cannot be attributed to any other tooth pathology [ 8 ] and it occurs mostly in canines and premolars [ 9 ]. When the tooth is preparing for a full crown, approximately 1.2 to 1.5mm is reduced for the proper contour of the crown and sufficient occlusal clearance [ 10 ]. Richardson *et al.* [ 11 ] stated that when a molar tooth is prepared, about 1-2 million tubules are uncovered in the oral cavity.

In recent decades, due to the introduction of new methods and the increase in sensitivity after cementation, desensitizing agents have been widely used [ 7 ]. GLUMA desensitizer is an adhesive system composed of 5% glutaraldehyde and 35% hydroxyl ethyl methacrylate (HEMA). It has been stated that the dentinal tubules are sealed by the reaction of glutaraldehyde with plasma proteins from dentinal fluid and decreased sensitivity [ 12 - 13 ]. 

Over the past decades, with advancements in laser technology, the utilization of lasers in dentistry has increased. Previous studies have shown the impact of lasers on the treatment of dentin hypersensitivity; the results differ as well as the irradiation parameters, wavelengths, and application techniques [ 14 ]. Several studies [ 14 - 16 ] showed diode laser could be effective on dentin hypersensitivity. Laser can stimulate the production of tertiary dentin at low energy densities, and some studies showed that laser could provoke dentinal melting and occlude dentinal tubules at higher energy densities, but it can cause thermal effects [ 14 , 17 - 18 ]. 

The goal of this study was to compare the effect of 940nm diode laser and GLUMA desensitizer on the bond strength of full-metal crowns cemented by self-adhesive resin cement.

## Materials and Method

In this *in vitro* experimental study, the least sample size was assumed to be ten samples in each of the groups (40). This calculation was in accordance with a study by Lawaf *et al.* [ 19 ] with Power Analysis Software PASS 11 assuming alpha=0.05, beta=0.2, the standard deviateon of 51.00 N and effect size of 0.61. The study was approved by the Ethics Committee of Tehran Azad University of Medical Sciences (IR.IAU.DENTAL.REC.1-397.034).

Thirty intact maxillary premolars among newly extracted teeth for orthodontic purposes were selected. For disinfection, the teeth were soaked in 0.1% thymol solution for 2 days. To clean the teeth, they were scaled with periodontal scalers [ 7 ].

**Figure 1:**
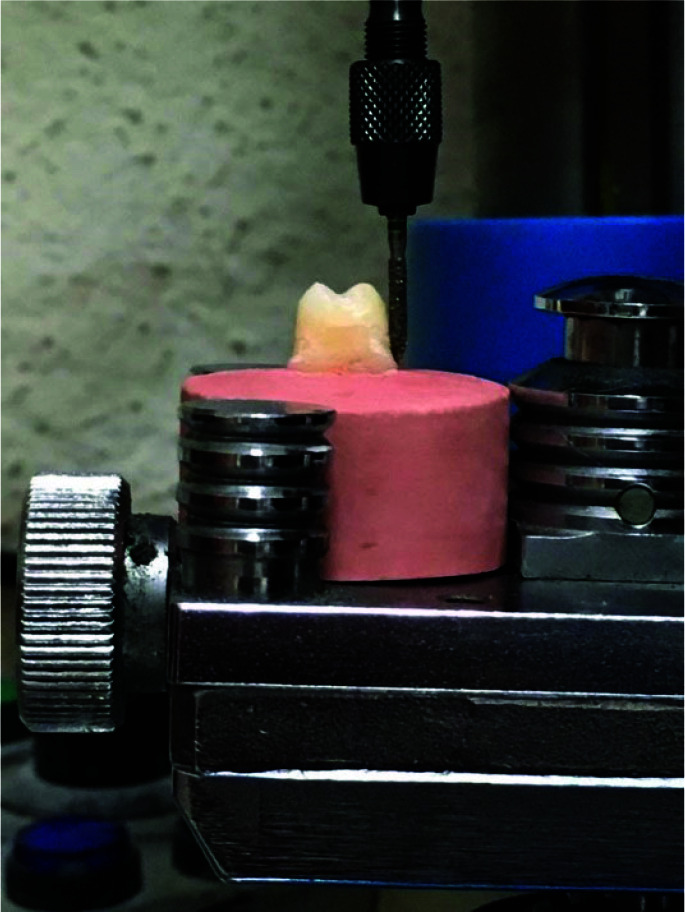
Preparation of a tooth with a milling machine

**Figure 2:**
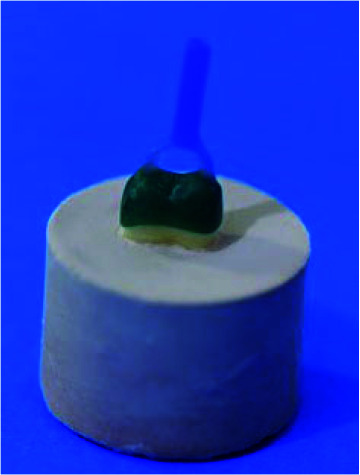
Wax model, a circlet was attached to the occlusal surface

The teeth were fixed on stone molds (Ariadent, Tehran, Iran) and prepared using a milling machine (Degussa, Germany) with a survey plane parallel to the survey platform. The teeth were positioned as vertically as possible to the surveyor's analysis rod. A round-end taper diamond bur (Dia-Burs, Mani Inc. Tochigi, Japan) was utilized for occlusal reduction, and a torpedo bur (Dia-Burs, Mani Inc. Tochigi, Japan) for axial reduction. Samples with a taper of 6°and a height of 4mm were used for the study. A final finish line with torpedo bur with width of 0.5-0.7mm was prepared above the cementoenamel junction (CEJ) (Figure 1). Then, the teeth were finalized and any sharpness was removed with abrasive strips [ 7 , 19 ]. The samples were scanned with a scanner (Amman Girrbach, Germany). The wax patterns with 0.5mm thickness were designed for each sample and fabricated on the teeth using a CAD/CAM (Ceramill Motion 2, Amman Girrbach, Germany) system [ 20 ]. A circlet was attached to the occlusal surface of the wax models (Figure 2), and then casted to use as a fixture for retention and testing in a universal testing machine (Zwick Z050; Roell Group, Ulm, Germany) [ 7 , 19 ]. The wax patterns were embedded using phosphate investment stone (Wirovest; Bego Corp., HanauWolfgang, Germany). The investment casting was used to make metal crowns. The crowns were put on the prepared teeth. The marginal fit and seating of the crowns were checked with a fit checker, explorer, and a magnifier. The crowns were finalized with metal finishing stones, burs and 50μm aluminum oxide particles were used to sandblast (Korox, BEGO, Germany) and put in an ultrasonic bath for cleaning (Ultraschall, Dentaurum, Germany) for 60 seconds.

Thirty samples were divided into different groups at random as: (1) Group A: Gluma desensitizer (Heraeus-Kulzer-Hanau, Germany) was used with the tip of the applicator on the samples and remained for 60 seconds. Compressed air was sprayed on the samples to remove the shiny surface, and then rinsed with water. (2) Group B: The samples received irradiation with a 940 nm diode laser (Dr-smile, Italy) with 0.5-watt power for 15 seconds continuously, three times with an interval of 24 hours. The laser was irradiated tangentially with a 1mm distance on the samples. (3) Group C: The samples received no intervention.

The RelyX U200 (3M ESPE, St. Paul, MN, USA) cement was prepared due to the manufacturer’s instruction to obtain equal thickness for all crowns. The crowns were charged with cement and inserted with strong finger pressure. An axial load of 5 kg was applied to samples for 10 minutes by a loading machine [ 19 , 21 ]. An explorer removed the excess cement. Then, to ensure the cement's curing, the crowns' margins were cured from each side by the light cure device (LED.D, Woodpecker, China) with 850 mW/cm2 light intensity for 20 seconds at a distance of 0.5 cm. Then all the samples were kept in an incubator (Kavosh Mega, Iran) at 37°C for 1 day. The teeth were taken out from the stone molds, and notches were made on their roots so they would not come out of the acrylic resin. Then the samples were mounted vertically in metal blocks 25× 25 ×30mm up to 2mm below the CEJ in self-cured acrylic resin (Acropars, Iran) [ 22 ]. 

**Figure 3:**
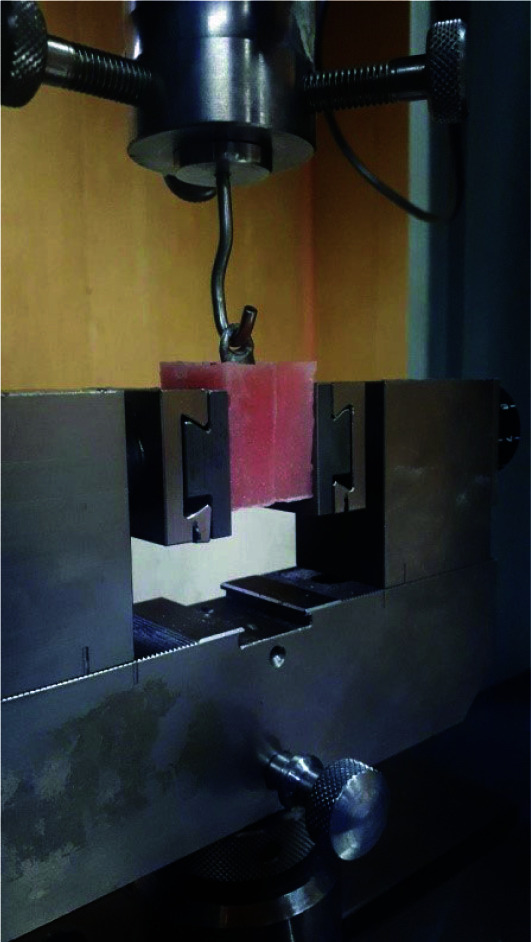
Load application to the samples

The retention test was carried out on a universal testing machine with a custom-made metal jig connected to it. A vertical tensile force was adjusted to each sample at 0.5mm/min till the crown detached from the tooth (Figure 3) [ 19 , 22 ]. After detachment of the crowns, the surfaces where the crowns came off were examined under a stereomicroscope (SMZ 800, Nikon, Tokyo, Japan) to discover failure mode [ 19 , 21 - 23 ]. The failure mode was categorized into three types: adhesive failure, with less than 25% of the bonding cement remaining on the tooth, cohesive failure, where more than 75% stayed intact; and mixed failure, in which the bonding cement ranged between 25% and 75% on the tooth. 

The results were established in percent and evaluated in four groups by Kolmogorov-Smirnov, ANOVA, Post Hoc Tukey Test, and independent t-test (SPSS version 20 software).

## Results

The standard deviation and coefficient of variation of the three study groups are shown in Table 1. 

**Table 1:** Results of t-test for comparison of tensile bond strength

Groups/ Tensile bond strength	Mean±SD	CV	*p* Value
Control group	138.17±40.81	29.5	0.000
Gluma group	318059±56.31	8.5	
940nm Diode Laser group	166.86±34.25	19.3	

The Kolmogorov-Smirnov test results in Table 2 show the bond strength data followed normal distributions for the three groups (Table 2) (*p*> 0.05).

**Table 2:** The results of Kolmogorov-Smirnov test

Group			Retention
Control group	N		10
	Normal Parametersa,b	Mean	138/1720
		Std. Deviation	40/81991
	Most Extreme Differences	Absolute	0/143
		Positive	0/143
		Negative	-0/110
	Test Statistic		0/143
	Asymp. Sig. (2-tailed)		.200c,d
Gluma group	N		10
	Normal Parametersa,b	Mean	318/5900
		Std. Deviation	56/31423
	Most Extreme Differences	Absolute	0/215
		Positive	0/215
		Negative	-0/125
	Test Statistic		0/215
	Asymp. Sig. (2-tailed)		.200c,d
940nm Diode Laser group	N		10
	Normal Parametersa,b	Mean	166/8640
		Std. Deviation	34/25155
	Most Extreme Differences	Absolute	0/160
		Positive	0/160
		Negative	-0/102
	Test Statistic		0/160
	Asymp. Sig. (2-tailed)		.200c,d

One-way ANOVA test analysis (Table 3) showed tensile bond strength had a significant difference in the study groups (*p*< 0.05). Table 4 shows the results of Post Hoc Tukey test. The results indicated that there is a significant difference in tensile bond strength between the GLUMA group (318.59±56.31 N) and the other groups (*p*< 0.05). In addition, there was not a significant difference between 940 nm diode laser and control groups (*p*> 0.05).

**Table 3:** The results of One-way ANOVA test

	Sum of Squares	Df	Mean Square	Mean Square	Sig.
Between groups	404853/092	4	101213/273	101213/273	0/000
Within groups	73353/432	45	1630/076		
Total	478206/525	49			

**Table 4:** The results of Post Hoc Tukey Test

(I) Group		Mean Difference (I-J)	Std. Error	Sig.	95% Confidence Interval	
					Lower Bound	Upper Bound
Control	Gluma	-180.41800*	18.05589	0/000	-231/7229	-132/6367
	Laser 940nm	-28.69200*	18.05589	0/512	-79/9969	145/0503
Gluma	Control	180.41800*	18.05589	0/000	129/1131	231/7229
	Laser 940nm	151.72600*	18.05589	0/000	100/4211	203/0309
940nm Diode Laser	Control	28.69200*	18.05589	0/512	-22/6129	79/9969
	Gluma	-151.72600*	18.05589	0/000	-203/0309	-100/4211

**Figure 4:**
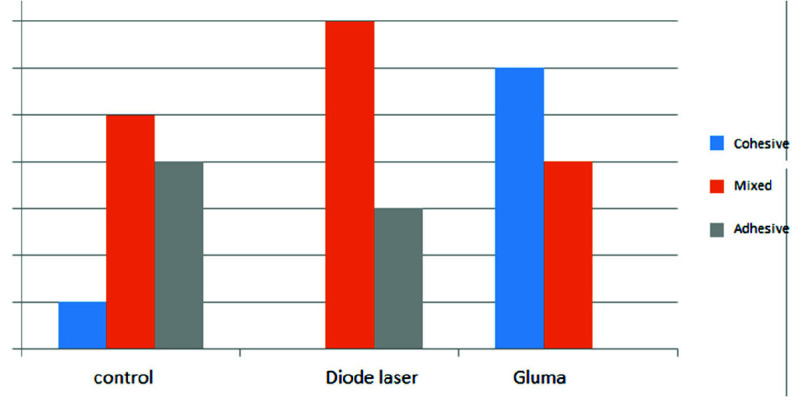
Modes of failures

In the GLUMA group, 6 samples showed cohesive failure, and the other samples showed mixed failure, with cement mostly left on the tooth. In the 940nm laser group, 7 samples had mixed failure and the rest had adhesive failure. The control group had 5 samples with mixed failure, 4 samples with adhesive failure and 1 sample with cohesive failure (Figure 4).

## Discussion

Post-operative dentin hypersensitivity is a significant problem most patients experience after tooth preparation for prosthodontic purposes [ 10 ]. Some studies [ 20 , 22 ] showed different types of desensitizing agents. Desensitizers create a barrier that protects tooth structure but it may prevent the micromechanical bonding of the cement and tooth. On the other hand, these desensitizing agents may affect the bond strength of cement, resulting in decreasing retention. However, it is important to pay attention to this point that crown retention depends primarily on the taper acquired and cement fills the space between tooth and crown to prevent microleakage [ 3 , 5 , 10 ].

In the present study, we compared the effect of 940nm diode laser and GLUMA desensitizer on the bond strength of full-metal crowns cemented by RelyX U200 self-adhesive resin cement. RelyX U200 has an acidic nature. It is partly hydrophilic when it is used but it turns into neutral and even hydrophobic after it sets. Accordingly, it can withstand water absorption finer and stay sturdy henceforward. It is not necessary to condition the dentin with a bonding agent [ 24 ].

This experimental study showed that the tensile strength in the GLUMA desensitizer group was significantly higher than in the other groups. Several studies [ 7 , 19 , 24 ] have explored the impact of desensitizing agents on the adhesion of dentine to different types of cement. 

Recently, various studies have examined the impact of desensitizers on adhesion with various types of cement. In some studies, [ 19 , 21 , 23 - 25 ] the use of the GLUMA desensitizer has been shown to have a positive effect on the bond strength of self-adhesive resin cement to dentin that was in accordance with the results of our study. Assadullah *et al.* [ 23 ] evaluated the effect of GLUMA and UltraSeal on crown retention using resinomer cement. They reported that applying GLUMA to resinomer cement increased retention. The majority of decementation was adhesive failure in all groups.

Mapkar *et al.* [ 21 ] assessed the impact of GLUMA and UltraSeal on crown retention using zinc phosphate cement. The cement they used differed from our study, but they reported the retention increased in the GLUMA group, and the difference was notable. The major failure mode was adhesive in groups.

Hernandez *et al.* [ 26 ] assessed the impact of GLUMA desensitizer, Desensibilize Nano P, and Soothe desensitizers on the shear bond strength of self-adhesive resin cement to dentin. They outlined that the instant and permanent shear bond strength of a self-adhesive resin cement dentin was not affected by the previous use of the desensitizers [ 26 ], which differs from our result. The contrast in results may be due to the different methodologies. They also used bovine incisors [ 26 ], but in our study, the specimens were maxillary premolars.

GLUMA desensitizer is an adhesive system that contains glutaraldehyde and hydroxyl ethyl methacrylate. Glutaraldehyde causes proteins and amino acids to coagulate in the tubules resulting in occluding of dentinal tubules and decreasing hypersensitivity. It also has a disinfecting effect [ 27 ]. Schupbach *et al.* [ 13 ] showed under scanning electron microscopy that numerous transverse septa appeared in the lumen of the dentinal tubules to a depth of 200 µm after GLUMA application. They hypothesized that the flow of dentinal fluid was affected by emergence of septum. Hydroxyl ethyl methacrylate could accelerate glutaraldehyde penetration into the tubules, where glutaraldehyde causes serum proteins to attach in the dentinal fluid and clogging of the tubules due to its high water solubility [ 13 ]. According to Schmidlin *et al.* [ 28 ] bond strength increased when glutaraldehyde was combined with HEMA. 

Glutaraldehyde/HEMA products include water so it can play as a rewetting agent. However, there is little information that HEMA could be in charge of increased bond strength [ 29 ].

In the past decades, laser has been used to decrease hypersensitivity [ 15 - 18 ]. In this study, we evaluated 940nm diode laser on bond strength of full metal crowns. Our study results showed that laser did not show a significant impact.

Morphological changes of dentin irradiated with laser can be seen under a scanning electron microscope, which depends on the frequency, application method and output power [ 30 ].

Although no similar study with a similar methodology was available that determined the impact of diode lasers on retention of full-metal crowns, we reviewed several studies [ 31 - 34 ]. Kasraei *et al.* [ 31 ] conducted the impact of diode laser on the microtensile bond strength of an etch-and- rinse adhesive to dentin. The results revealed that adhesive failures were more common in all groups [ 31 ]. Although the methodology differed from our study, they reported that irradiating the dentin surface with a 940 nm diode laser after using adhesive and before curing could increase the bond strength of the composite to the dentin [ 31 ]. Laser irradiation after using the adhesive can enhance the quality of the hybrid layer, which can lead to higher bond strength. Laser irradiation increases the temperature, which can improve adhesive penetration [ 31 ]. 

In addition, high temperatures can raise the degree of conversion of adhesive penetrating the dentin [ 32 ].

Mubaraq *et al.* [ 33 ] assessed the effect of desensitizing by Er:Cr:YSGG laser on shear bond strength. They reported that laser interaction with dentin causes water absorption and conversion to steam, and steam expansion causes microexplosion. This explosion causes dentin debris to occlude dentinal tubules. They concluded that dentin surface roughness caused by laser irradiation increases the shear bond strength. This study demonstrated that laser irradiation could lead to dentin surface roughness, which in turn increases shear bond strength. This finding is significant because it suggests that laser treatment may alter the surface characteristics of dentin, potentially enhancing the bond between the tooth and the crown. By referencing this study, we aim to provide a broader context for understanding how laser technology can impact dental procedures and outcomes. While our study focused on a different type of laser and examined its effects on crown retention specifically, the findings of Mubaraq *et al.* [ 33 ] contribute to the overall understanding of how laser treatment can influence dental bonding mechanisms.

Souza-Gabriel *et al.* [ 34 ] assessed the effect of Er: YAG and diode lasers on the shear bond strength. They concluded diode laser (980nm, 1.5 W) showed an unfavorable effect on bond strength. Adhesive was more common type of failure in all groups. They explained this laser was not able to eliminate the smear layer and affected the adhesion of adhesive system. In our study, we examined the effect of a 940nm diode laser on the bond strength of full-metal crowns. Souza-Gabriel *et al.* [ 34 ] investigated the impact of both Er:YAG and diode lasers, including a diode laser with slightly different specifications (980nm, 1.5 W), on shear bond strength. Although our studies differ in laser types and parameters, both explore the influence of laser technology on dental bonding. Referencing Souza-Gabriel *et al.* [ 34 ] provides insights into how different laser parameters may affect bond strength and underscores the importance of understanding laser-assisted dental procedures' underlying mechanisms. Their findings highlight the need to consider laser parameters carefully in enhaning bond strength in dental restorations. 

Due to the limitations of this study, we recommend further studies with larger sample sizes, different desensitizing agents, and laser application with different device settings to robustly evaluate the efficacy and optimize the clinical application of these interventions and to enhance the depth of understanding and broaden the scope of research in this area.

## Conclusion

The study compared the impact of GLUMA desensitizer and 940nm diode laser on the bond strength of full-metal crowns. GLUMA desensitizer notably improved bond strength, while the diode laser showed no significant effect. This highlights the critical role of selecting suitable desensitizers for enhancing crown retention. Further research with broader sample sizes and diverse methodologies is necessary to comprehensively understand the effects of various desensitizers and laser settings on crown retention in clinical practice.
